# Bounded Rationality and Voting Decisions over 160 Years: Voter Behavior and Increasing Complexity in Decision-Making

**DOI:** 10.1371/journal.pone.0084078

**Published:** 2013-12-31

**Authors:** David Stadelmann, Benno Torgler

**Affiliations:** 1 Faculty of Law, Business and Economics, University of Bayreuth, Bayreuth, Germany; 2 CREMA – Center for Research in Economics, Management and the Arts, Basel, Switzerland; 3 School of Economics and Finance, Queensland University of Technology, Brisbane, Australia; 4 EBS Universität für Wirtschaft und Recht, Oestrich-Winkel, Germany; Durham University, United Kingdom

## Abstract

Using a quasi-natural voting experiment encompassing a 160-year period (1848–2009) in Switzerland, we investigate whether a higher level of complexity leads to increased reliance on trusted parliamentary representatives. We find that when more referenda are held on the same day, constituents are more likely to refer to parliamentary recommendations when making their decisions. This finding holds true even when we narrow our focus to referenda with a relatively lower voter turnout on days on which more than one referendum is held. We also demonstrate that when constituents face a higher level of complexity, they follow the parliamentary recommendations rather than those of interest groups. “Viewed as a geometric figure, the ant’s path is irregular, complex, hard to describe. But its complexity is really a complexity in the surface of the beach, not a complexity in the ant.” *(* [*1*] *p. 51)*

## Introduction

As part of the recently revived commitment to *correctly* describing or prescribing human economic affairs, social science has been aiming to achieve a single, widely-accepted, and precise behavioral theory of choice. Simultaneously, increased interest in the idea that human rationality is bounded has generated a need for a theoretical framework incorporating more realistic models of human actors. As noted by Herbert Simon, a pioneer of this concept [2]–[3], the principle of bounded rationality recognizes limitations of the human mind in solving complex problems [1]. Indeed, Kahneman’s Nobel Prize exploration of the biases that permit decision-making under complex conditions was an attempt to map bounded rationality [4].

Almost a century ago, John Maurice Clark proposed ideas about decision-making that are consistent with the concept of bounded rationality and satisfaction [5]. Most particularly, Clark stressed that decision making involves a level of attention and effort that cannot be sustained for long because of limited cognitive capacity. He linked this discussion to such works as Cooley’s *Human Nature and Social Order*, which describes humanity’s tendency to engage in a mechanical search for (among other things) an accepted personal authority. Clark’s observations reflect the focus of our research and the aim of this paper: we empirically investigate whether a higher level of complexity leads to an increased reliance on simple rules of thumb. To achieve this aim, we explore whether humans rely on trusted representatives as a rule of thumb when many problems demand simultaneous concentrated attention, a topic on which, to our surprise, there is little empirical evidence. Specifically, we thereby test bounded rationality by analyzing constituents’ real voting behavior when parliamentary members are the trusted representatives.

A rich model of voting behavior requires a good understanding of voters’ attention and beliefs [6]. Switzerland offers a particularly useful case study because the Swiss parliament provides ex-ante voting recommendations for referenda, a standard method of information provision that enhances its image as a trusted consultant. Because in most modern democracies information provision is delegated to politicians, interest groups, the government, and/or professional information gatherers [7]–[8], constituents might be inclined to follow the recommendations of their elected parliament, which is supposed to represent them. Hypothesizing that voting decisions will be influenced by parliamentary leaders who inform voters about the issues [9], we identify the effect of public parliamentary voting recommendations on constituents’ *real choices* in referenda (i.e., on the revealed preferences of the constituents) over 160 years (from 1848–2009) using parliamentary recommendations as the observable and measurable variable. Given that citizens of the Nordic countries, Switzerland, and Luxembourg tend to report the highest levels of trust in their national parliaments and have the highest level of overall trust in political institutions [10], it is not unreasonable to suggest that Swiss constituents perceive members of Switzerland’s parliament as trusted representatives.

The Swiss parliament itself comprises two houses: the National Council (*Nationalrat)*, and the Council of States (*Ständerat*), which represent 26 parliamentary electoral districts (cantons or subnational jurisdictions). Because Switzerland’s parliamentary system is one of direct democracy through referenda, not only can citizens from the different constituencies challenge any law passed by parliament and propose initiatives, but a referendum is mandatory whenever a parliamentary legislative proposal aims to change the constitution. Citizens are assisted in their voting decisions by political information provided ex-ante by the government [11]–[12]. Hence, referendum results in Switzerland not only determine policy outcomes, they also reflect citizens’ preferences for these outcomes. More precisely, referenda allow the majority to rank the policy outcomes that will be generated by the proposed laws against the status quo [13]–[15]. Referenda thus present dichotomous results that indicate what is preferred by the majority and, consequently, by the median constituent [15]–[16].

Because our study analyzes the outcomes of different referenda which are held on the same day, we must take into account that people have a narrow capacity for simultaneous attention to different pieces of information [17]. In other words, humans can only deal with a limited number of problems at one time. We model the information complexity in our setting in two distinct ways: (1) by differentiating referenda on days with more referenda from those on days with only one referendum and (2) by identifying referenda that receive a relatively lower turnout in a constituency on days with more than one referendum. We expect that when complexity is high–that is, several voting decisions are asked for on the same day or turnout for the referendum is low–voters will be more likely to follow parliamentary recommendations as a simple rule of thumb.

We recognize that the changing shape of the decision-making environment may influence human goal-directed behavior over time [6]; for example, over the long sample period, citizens have grown up in very different social environments, with different knowledge, information and social norms. Nonetheless, not only does our institutional setting remain constant for the whole period analyzed but even in complex situations the same basic decision-making rule may apply, thereby enabling identification of any common invariant of human behavior in decision-making.

To the best of our knowledge, no studies currently exist that explore bounded rationality in such a real world context over such a long time span. Hence, our setting is particularly well suited for deriving empirical insights on the *consequences* of bounded rationality in real voting situations. Moreover, by using voter turnout, we can directly measure which issues receive more attention, i.e., we can identify voters’ *focus of attention*, and offer a clear explanatory heuristic for the decision process mechanism. Hence, by employing simple rules of thumb to identify satisficing decision outcomes [18], we can provide answers not only on *which* decisions are made but also on *how* they are made, thereby contributing to expand the knowledge of decision-making processes with empirical evidence on *procedural rationality*. Another advantage of the Swiss case is that the net cost of obtaining information is less of a problem because parliamentary recommendations are readily available in a booklet/pamphlet sent to voters prior to a referendum. In fact, since 1877 the federal council has been required by law to issue information detailing the legislative proposal submitted. In other words, most of the information needed for decision making is simply placed directly into the voters’ hands (Figure S1). We therefore derive and empirically test the following hypothesis: *When more referenda are held on the same day (thereby increasing decision-making complexity), voters will, ceteris paribus, be more likely to refer to outside recommendations from the parliament in their decision making*.

### Data

The availability of referendum voting data over a long time period (from 1848 when the first national referendum was held, until the beginning of 2009) stems from the stability of Switzerland’s institutional environment. Hence, our dataset comprises 555 federal referenda including counterproposals to citizens’ initiatives. Because these frequent referenda present constituents with dichotomous, inexpensive decision choices, they fulfill a central requirement for valid identification of the use of rules of thumb [19].

**Figure 1:**
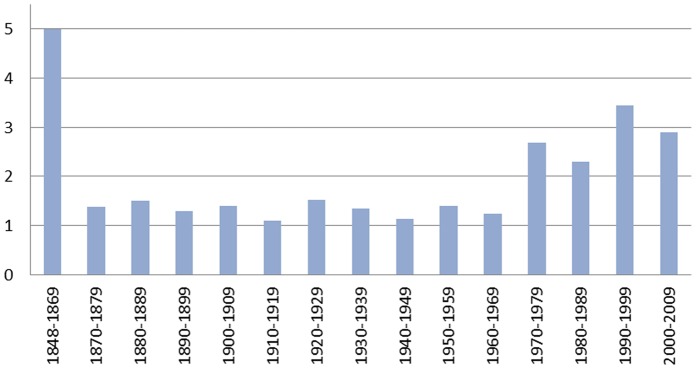
Average number of referenda per voting day in different periods. Since the first federal referendum in 1848, voters have usually been given the chance to go to the polls between one and four times a year to make decisions on a minimum of one and a maximum of nine distinct federal referenda issues.

**Figure 2:**
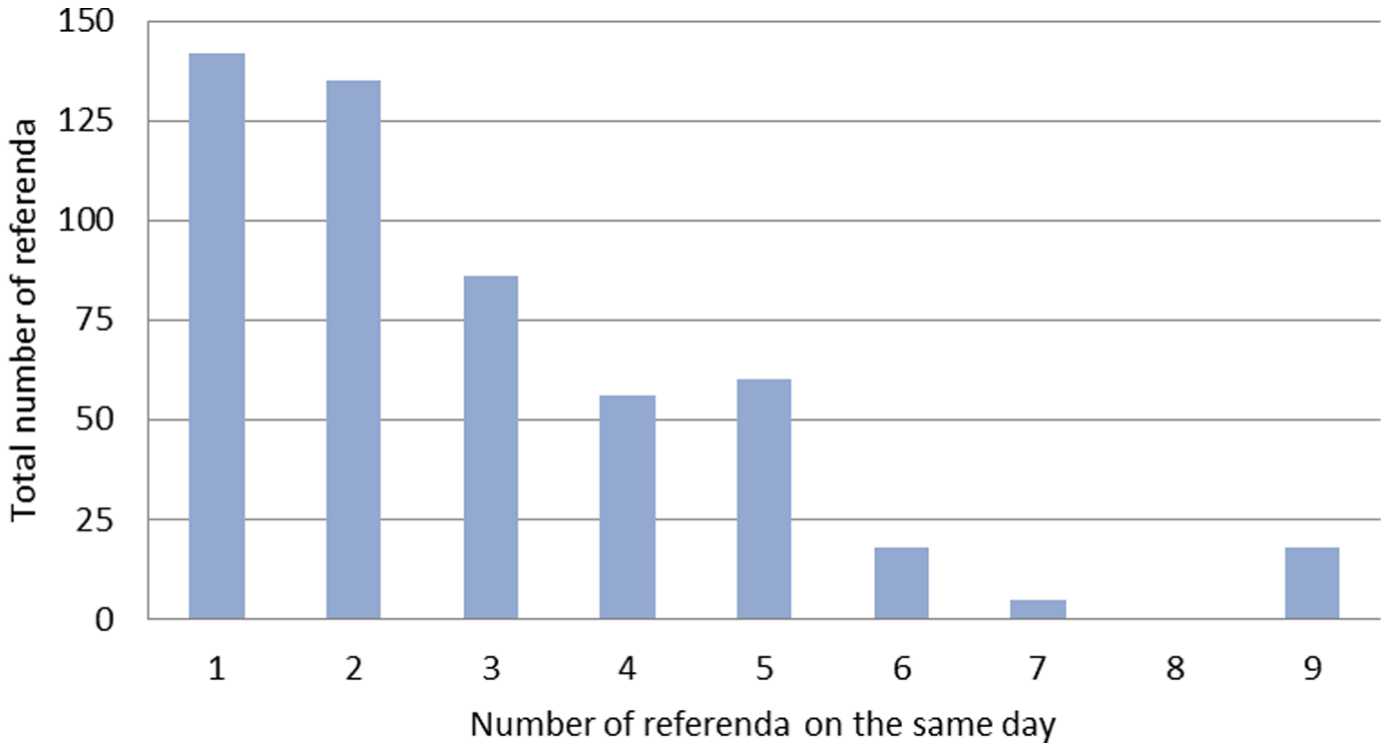
Histogram of number of referenda on the same day from 1848 to 2009. The x-axis indicates the number of referenda on the same day, and the y-axis shows the total number of referenda (without counterproposals) for the respective category. Although voters usually decide on five or fewer referenda on the same day, since 1848, there have been occasions on which nine referenda decisions were made. Although seven referenda decisions were made on May 17, 1992, there is no record of eight referenda decisions on a single day.

As Figure 1 shows, the average number of referenda per voting day remained stable in all decades from 1870 until after World War II and then increased only slightly (Figure S2 shows the total number of referenda from 1848 to 2009). In Figure 2, we present a histogram with the number of referenda per day over our sample period excluding counterproposals, which are omitted because they are always presented with the original referendum, making the referendum a multiple decision between the status quo, the proposed initiative, and the counterproposal. During the current and past two centuries, there have been over 125 voting days on which exactly one referendum was held; on the remaining days, two or more referenda were on the ballot. All additional variables employed in the analysis, their sources, and a number of descriptive statistics are provided in the supporting information (Table S1).

## Results

To determine the probability that a constituency will accept a referendum, we use a logit model in which the dependent variable is a constituency’s acceptance of the referendum. For each referendum, parliament either recommends a “yes” or a “no” vote and from this we construct the variable *Parliament suggests YES*. We include one of our two measures for complexity: *Number of referenda on the same day* and *Low turnout referendum*. As referenda are preceded by a public discussion, such a discussion is more difficult for constituents to follow if the number of referenda on the same day increases. Recognizing that constituents are more likely to cast a vote for a referendum about which they are better informed, while constituents with little information may still vote but rely more on parliamentary recommendations, our second measure for complexity directly identifies voters’ focus of attention by using the percentage of voter turnout on days with more than one referendum.

An interaction term between *Parliament suggests YES* and the respective complexity measures allows us to identify whether the voters are applying simple decision-making rules in complex situations. We also calculate discrete effects for the influence of the parliamentary recommendation and the interaction terms, while holding all other variables fixed at their medians [20], and report robust clustered standard errors.

Specification (1) in Table 1 shows that the pseudo R^2^ is high for a logit model (and indeed for this type of analysis) even though the model includes no other controls apart from constituency fixed effects (cantonal dummies). Because both interaction variables are exogenous to constituent referenda choices, the interaction term indicates the causal influence of parliament’s recommendation in more complex voting situations; that is, when voters are faced with more referenda on the same day. Thus, any variable not present in the estimations can only be characterized as omitted if it influences the interaction term directly, a contingency we control for in subsequent estimates and refinements. As shown in Table 1, the interaction term is highly statistically significant at the 1% level (robust standard errors clustered for referenda) with a positive sign. Thus, when there are more referenda per day, the parliamentary influence on constituent decisions is larger. Specifically, the discrete effects show that when only one single referendum is proposed, constituents are 45.95 percentage points more likely to vote yes when parliament recommends to vote yes, while the interaction term indicates that this effect increases by 9.24 percentage points when the number of referenda increases from one to three (the median on days with more than one referendum).

**Table 1:** Parliamentary influence on constituent referenda choices.

	Identification: Number of referendaon the same day			Identification: Low turnout referendum	
	(1)	(2)	(3)	(4)	(5)
Years covered	1848–2009	1848–2009	1884–2009	1884–2009	1884–2009
Parliament suggests YES	1.7323***(0.2742)	1.7403***(0.2902)	1.7213***(0.2922)	2.4834***(0.1212)	2.5485***(0.1227)
Parliament suggests YES * Number of referenda **on the same day**	0.2641*** **(0.0875)**	0.3103*** **(0.0963)**	0.3068*** **(0.0973)**		
Number of referenda on the same day	−0.1950**(0.0777)	−0.1793**(0.0827)	−0.1832**(0.0843)	0.0574***(0.0107)	0.0748***(0.0143)
Parliament suggests YES * Low turnout referendum				0.4010*** **(0.1113)**	0.2956*** **(0.1140)**
Low turnout referendum				−0.1201(0.1013)	−0.0689(0.1014)
Counterproposal		−0.2083(0.3073)	−0.2979(0.3110)		−0.4030***(0.0514)
Turnout			−1.9731***(0.4562)		−1.4259***(0.2443)
Constituency fixed effects	YES	YES	YES	YES	YES
Decade fixed effects	NO	YES	YES	NO	YES
DE Parliament suggests YES	45.9540	46.7379	46.5972	53.3954	53.5781
DE Parliament suggests YES * Number of referendaon the same day	9.2357	10.8994	10.9059		
DE Parliament suggests YES * Low turnout referendum				7.1800	5.5651
Clustering	Referendum	Referendum	Referendum	Constituency	Constituency
Sample restriction				>1 referendum	>1 referendum
Pseudo R2	0.2996	0.3312	0.3505	0.3485	0.3671
Brier	0.1925	0.1853	0.1808	0.1812	0.1765
No. obs.	14127	14127	13502	9961	9961

**Notes:** The dependent variable for all logit estimations is *Constituency accepts referendum*. Robust clustered standard error estimates for referenda (columns 1–3) and constituencies (columns 4–5) are reported throughout the table. An intercept is always included. *DE* denotes the discrete effects in the predicted probability. The discrete effects for *Parliament suggests YES* * *Number of referenda on the same day*, and *Parliament suggests YES* * *Low turnout referendum* represent changes in percentage points for the coefficients when all other variables are evaluated at their median values and the *Number of referenda on the same day* changes from one to three (columns 1–3), and *Low turnout referendum* changes from zero to one (columns 4–5). ***, **, and * indicate a mean significance level of below 1%, between 1 and 5%, and between 5 and 10%, respectively.

Specification (2) includes a dummy for counterproposals to ensure that the observed effects are not a consequence of different reactions to parliament when there is more than one referendum and one is a counterproposal. We also introduce decade dummies to capture technological and environmental changes that have reduced the transaction costs of information, potential changes in voter characteristics (e.g., average level of education), and/or a shift to more referenda over time. Other changes that justify the use of such time dummies include the granting women the right to vote at the federal level in 1971 and the emergence of the new canton of Jura. Including the counterproposal variable and decade dummies, however, has barely any effect on our key variables: the coefficient of the interaction term remains positive and statistically significant with a comparable discrete effect (in fact, the quantitative effect is even a little larger).

Specification (3) then restricts the sample to the 1884–2009 period to reflect the fact that voter turnout data are only consistently available at the cantonal level from 1884 (before there was no systematic registration of either the electorate or the number of valid votes in all constituencies). Turnout not only measures voters’ level of interest or how well informed they are, it also covers such aspects as environmental conditions (e.g., weather). Controlling for this factor is important because voters might stay away if they are not well informed, particularly in situations where there is more than one referendum. Our results, however, indicate not only that turnout has no effect on the interaction term but that the discrete effect actually increases slightly to almost 11 percentage points. In other words, when more referenda are held on the same day, the influence of parliament’s recommendations on constituents’ real choices is significantly higher. The base effect of turnout is also negative, which may indicate that voters come to the polls if they disagree (status quo bias). This finding, however, should not affect our quasi-experimental setting with respect to the effect of parliament’s recommendations in more complex voting situations and constituents following a rule of thumb.

To fulfill our second aim (directly identifying the focus of attention), specifications (4) and (5) include an interaction term based on referenda with a relatively lower turnout within a constituency on days with more than one referendum (*Low turnout referendum*) instead of focusing on the number of referenda on the same day. The data sample is thus restricted to cases with more than one referendum per day. Because low turnout referenda are identified at the cantonal level, we report robust standard errors clustered by constituency in recognition of the likelihood that observations in the same constituency are not independent. In line with the previous results, the interaction term in specification (4) is positive and statistically significant and the discrete effect, at 7.18 percentage points, is large. To ensure that the effects from the first identification strategy do not overlap with those from the low turnout referenda, we also control for the *Number of referenda on the same day*. In line with the earlier analysis, we include the counterproposals, check for the effects of turnout itself, and include decade fixed effects (in specification 5). Throughout all the estimations reported, the interaction term is statistically significant at the 1% level with a discrete effect between 5.56 and 7.18 percentage points. All subsequent estimations include cantonal and decade fixed effects.

Next, we refine our specifications as follows: in specification (1) of Table 2, we analyze the interaction term using the dummy variable *More than one referendum* instead of *Number of referenda on the same day*. Again, the interaction is positive and significant, with a discrete effect over 9 percentage points. To expand our analysis of interaction terms, in specifications (2) and (3), we concentrate on days with more than two or more than three referenda. In all cases, the interaction term is significantly positive, and the discrete effects increase with complexity, thereby confirming the effects noted previously. Importantly, the interaction term also remains significantly positive when we apply our second identification strategy for complexity (*Low turnout referendum*) in specifications (4) and (5) restricting the sample accordingly.

**Table 2:** Refinement using number of referenda on the same day.

	Identification: Number of referendaon the same day			Identification: Low turnout referendum	
	(1)	(2)	(3)	(4)	(5)
Years covered	1848–2009	1848–2009	1848–2009	1884–2009	1848–2009
Parliament suggests YES	2.1695***(0.2763)	2.2591***(0.2095)	2.3493***(0.1986)	2.7116***(0.1709)	2.5194***(0.1566)
Parliament suggests YES * **More than one referendum**	0.5883* **(0.3458)**				
More than one referendum	−0.4734(0.3164)				
Parliament suggests YES * **More than two referenda**		0.7403** **(0.3628)**			
More than two referenda		−0.7052**(0.3250)			
Parliament suggests YES * **More than three referenda**			0.8871** **(0.4141)**		
More than three referenda			−0.3194(0.3578)		
Parliament suggests YES * **Low turnout referendum**				0.5391*** **(0.1538)**	0.8113*** **(0.1964)**
Low turnout referendum				−0.1951(0.1260)	−0.2158(0.1582)
Number of referenda on the same day				0.1740***(0.0229)	0.0850**(0.0366)
Counterproposal	−0.2180(0.3051)	−0.2126(0.3056)	−0.1930(0.3080)	−0.7686***(0.0785)	−0.4511***(0.1032)
Turnout				−3.1623***(0.5265)	−3.9928***(0.8108)
Constituency fixed effects	YES	YES	YES	YES	YES
Decade fixed effects	YES	YES	YES	YES	YES
DE Parliament suggests YES	49.1786	51.0306	46.4629	53.4686	53.0404
DE Parliament suggests YES *More than one referendum	9.3615				
DE Parliament suggests YES *More than two referenda		10.5967			
DE (Parliament suggests YES) *(More than three referenda)			15.7919		
DE (Parliament suggests YES) *(Low turnout referendum)				9.1936	14.0232
Sample restriction				>2 referenda	>3 referenda
Clustering	Referendum	Referendum	Referendum	Constituency	Constituency
Pseudo R2	0.325	0.3272	0.3307	0.4539	0.494
Brier	0.1862	0.186	0.1851	0.1551	0.1459
No. obs.	14127	14127	14127	6329	4015

**Notes:** The dependent variable for all logit estimations is *Constituency accepts referendum*. Robust clustered standard error estimates for referenda (1–3) and constituencies (4–5) are reported throughout the table. DE = discrete effect in the predicted probability (see Table 1 and text for details). ***, **, and * indicate a mean significance level of below 1%, between 1 and 5%, and between 5 and 10%, respectively.

Because interest groups may also give voting recommendations that can directly influence referenda outcomes [21], our next step is to investigate their effect in Table 3 using official interest group voting recommendations, which were first officially collected in 1945. For this time period, we use data for seven groups whose interests are, respectively, business, (*Zentralverband schweizerischer Arbeitgeber-Organisationen, Economiesuisse,* and *Schweizerischer Gewerbeverband*), unions (*Schweizerischer Gewerkschaftsbund* and *Travail.Suisse* which merged with *Vereinigung schweizerischer Angestelltenverbände* in 2002), and farmers (*Schweizerischer Bauernverband*). Although other interest groups and regional organizations exist, these seven are arguably the most important and have the highest number of active participants.

**Table 3:** Parliamentary influence on constituent referenda choices in the presence of interest group recommendations.

	Identification: Number of referendaon the same day				Identification: Low turnout referendum	
	(1)	(2)	(3)	(4)	(5)	(6)
Years covered	1945–2009	1945–2009	1945–2009	1945–2009	1945–2009	1945–2009
Parliament suggests YES	0.8381**(0.4152)	0.6415(0.4584)	0.9773**(0.4356)	0.7212(0.4841)	1.8958***(0.1579)	1.8679***(0.1711)
Parliament suggests YES * **Number of referenda on the same day**	0.3914*** **(0.1065)**	0.4495*** **(0.1193)**	0.3862*** **(0.1148)**	0.4641*** **(0.1303)**		
Number of referenda on the same day	−0.1971**(0.0822)	−0.1976**(0.0824)	−0.1828**(0.0919)	−0.1836**(0.0920)	0.0811***(0.0150)	0.1220***(0.0197)
Parliament suggests YES * **Low turnout referendum**					0.4466*** **(0.1368)**	0.6996*** **(0.1566)**
Low turnout referendum					−0.1440(0.1052)	−0.3484***(0.1201)
All interest groups suggest YES * **Number of referenda on the same day**		−**0.1151** **(0.1195)**		−**0.1505** **(0.1246)**		
All interest groups suggest YES * Low turnout referendum					−**0.0234** **(0.1128)**	−**0.0310** **(0.1183)**
All interest groups suggest YES	1.9740***(0.4395)	2.3426***(0.5870)	1.9209***(0.4462)	2.4120***(0.6146)	1.8365***(0.1899)	1.8812***(0.2105)
Interest groups divergent	1.1326***(0.4042)	1.1520***(0.4107)	1.2477***(0.3980)	1.2716***(0.4068)	1.0542***(0.1435)	1.3126***(0.1738)
Control for Counterproposal and Turnout	YES	YES	YES	YES	YES	YES
Cantonal fixed effects	YES	YES	YES	YES	YES	YES
Decade fixed effects	YES	YES	YES	YES	YES	YES
DE Parliament suggests YES	19.2457	16.3342	19.0234	15.4688	26.1650	21.1723
DE Parliament suggests YES *Number of referenda on the same day	12.2643	14.5481	11.4972	14.3397		
DE Parliament suggests YES *Low turnout referendum					7.8740	8.8518
Sample restriction	> = 1 interest group	> = 1 interest group	> = 2 interest group	> = 2 interest group	> = 1 interest group & >1 referendum	> = 2 interest groups & >1 referendum
Clustering	referendum	referendum	referendum	referendum	constituency	constituency
Pseudo econdR2	0.4325	0.4334	0.4269	0.4286	0.429	0.4258
Brier	0.1632	0.163	0.1652	0.1648	0.1625	0.1643
No. obs.	10201	10201	8932	8932	8385	7516

**Notes:** The dependent variable for all logit estimations is *Constituency accepts referendum*. Robust clustered standard error estimates for referenda (1–4) and constituencies (5–6) are reported throughout the table. DE = discrete effect in the predicted probability (see Table 1 and text for details). ***, **, and * indicate a mean significance level of below 1%, between 1 and 5%, and between 5 and 10%, respectively.

In this analysis, we evaluate only those referenda for which at least one interest group made an official voting recommendation and construct dummies that identify whether the interest group recommendations are divergent or whether all interest groups suggested a yes vote. As Table 3 shows, if interest groups suggest a yes vote, not only does the probability of constituents accepting the referendum increase but the coefficient of the variable *Parliament suggests YES* decreases (see specification 1). The goodness of fit of the estimation increases compared to earlier estimates which suggests that interest groups recommendations are an important factor explaining voting decisions. We, however, are interested in exploring whether, in more complex situations, voters tend to rely more on parliamentary recommendations or whether interest group recommendations play a larger role. We find that the interaction term between the parliamentary suggestion and the number of referenda on the same day remains positive, significant and of similar size compared to earlier specifications (specification 1), whereas the interaction between interest group recommendation and the number of referenda on the same day is not statistically significant (specification 2). Thus, once complexity increases, constituents are more likely to follow parliament than the interest groups. Such a finding remains robust even after we restrict our sample to those referenda on which at least two interest groups expressed an opinion (specifications 3 and 4). Likewise, in specifications (5) and (6), which focus on the alternative complexity variable *Low turnout referendum*, the interaction term Parliament suggests YES * Low turnout referendum is statistically significant and positive, while the coefficient of the interaction term for these referenda and interest groups is again insignificant. In sum, the results remain fully robust even when controlling for interest groups or when the method for identifying complexity is changed (i.e., we identify the voters’ focus of attention directly by measuring the percentage of voter turnout). Overall, as complexity increases, citizens are more likely to listen to elected politicians as trusted representatives than to interest groups.

We report the results of additional robustness tests in the supporting information. The first section takes into account that the government’s executive council (the federal council) also frequently makes recommendations on referenda, which means that constituents may not only be influenced by parliament (Table S2, columns 1–3). We also take account of minority positions in parliament (Table S2, columns 4–6). The second section explores different time periods in recognition of the post-1945 tendency to hold more referenda on one day (Table S3). The third section reports rolling regressions that omit one constituency at a time (Table S4). All results support our earlier findings.

## Discussion

To determine whether a higher level of complexity leads to an increased reliance on trusted representatives in real voting decisions, a topic on which there is surprisingly little empirical evidence, we exploit a natural setting of voter practices in Switzerland, whose institutional environment has been stable since 1848. The dataset for this unique setting encompasses 160 years (1848–2009) of information on real voting behavior in referenda. Our results suggest that when several simultaneous problems demand concentrated attention, humans follow a simple rule of thumb by relying on trusted representatives.

First, to test our prediction that holding more referenda on the same day means a greater likelihood that constituents will seek outside help, we use the number of these referenda to identify parliament’s influence on constituent voting behavior in the face of simultaneous problems that demand concentrated attention. We find evidence that parliamentary influence is indeed greater when there are multiple referenda than when there is only one referendum per day. Second, recognizing that on days with two or more referenda, constituents incur the same costs in turning up for the first as for the second or third referendum on the same day, we use turnout differences within constituencies to identify which referendum on a given day attracted the most attention. The strength of this empirical design is that we can use the percentage of voter turnout to directly identify voters’ *focus of attention* when decisions are to be made on multiple aspects. Not only are the quantitative effects large, but they remain consistently so throughout our robustness tests and refinements. We also control for the effects of interest groups and show that constituents are more likely to listen to parliament for their recommendations than to interest groups. Thus, voters are, ceteris paribus, more likely to refer to outside recommendations from the parliament in their decision when the level of complexity increases.

If politicians deliberately bundle referenda they want pass, then our findings of the increased likelihood of having a referendum supported on a day with more referenda would represent a lower bound estimate for the influence of parliament. Whether or not it is the case that politicians bundle more referenda on a single day to increase the power of their recommendations, our results provide solid evidence that constituents do follow a rule of thumb in complex and attention demanding situations.

Certain groups of voters might perceive specific issues as more complex and rely more on parliamentary recommendations to decide on them. Our study does not focus on referenda content. However, the empirical identification and exploration of rules of thumb necessitates a method for identifying *environmental* complexity that is well suited to frequent repetitions [19]: More referenda on the same day will dilute the attention of voters and thus make decisions relatively more complex. Because constituents face a repeated decision environment in which referenda occur frequently and the complexity level is approximated by the number of referenda on the same day, we are able to directly identify which cases attract less voter attention (i.e., which referenda have a relatively lower turnout).

Overall, the empirical analysis suggests that, once the voting task is held constant over a long period, a bounded rationality framework may shed light on *how* people vote when complexity of the decision environment changes. The simplicity of our setting allows us to identify the effect of parliamentary voting recommendations on constituents’ *real choices*. Most particularly, we identify an invariance in voting behavior that almost resembles a law of *qualitative* structure [22]. We also observe a tendency for voters to follow the rule of thumb or heuristic that dictates reliance on trusted representatives’ advice when many problems demand concentrated attention simultaneously. As this behavior has remained robust since the middle of the 19^th^ century despite environmental changes, it implies that a simple choice mechanism underlies a substantial number of observed choices. Our empirical analysis thus provides valuable insights into how humans use limited computational capacity to handle differences in information complexity, a type of complexity always present in modern societies but particularly so in politics where managing the distribution of scarce attention resources among competing agenda is vital to policy formation [23].

## Methods

The dependent variable 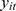 in the tables is constituency acceptance of the referendum. We perform a logit analysis of the probability of such acceptance based on the following generic estimation equation 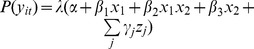. *λ* is the logistic function 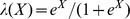, 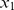 denotes parliament’s recommendation, 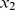 reflects either the number of referenda on the same day (our first measure of a complex environment) or referenda with a relatively lower turnout on the same day (our second measure of a complex environment). The coefficient 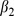 of the interaction between 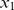 and 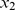 reflects the influence of parliament’s recommendation in a complex environment on the probability of a constituency accepting a referendum. If constituents are boundedly rational, they will apply the simple rule of thumb and follow parliament in more complex situations. Thus, we expect that 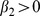. Finally, the coefficients 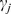 account for the effect of additional control variables, turnout in particular but also constituency and decade fixed effects.

## Supporting Information

Figure S1 Extract of federal legislation in 1877.
(DOC)

Figure S2 Total number of referenda from 1848 to 2009.
(DOC)

Table S1 Data description and sources.
(DOC)

Table S2 Parliamentary influence on constituent referenda when government is neutral and members of parliament offer divergent suggestions.
(DOC)

Table S3 Robustness tests with different periods – Influence of Parliament on constituents’ choices in referenda.
(DOC)

Table S4 Rolling regression – Parliamentary influence on constituent referenda choices.
(DOC)
