# Supplementary material for: Bounded Rationality and Voting Decisions over 160 Years: Voter Behavior and Increasing Complexity in Decision-Making
Source: PLoS One. 2013 Dec 31;8(12):e84078. doi: 10.1371/journal.pone.0084078 (PMC3877213; doi:10.1371/journal.pone.0084078)
Supplement: Figure S1 — Extract of federal legislation in 1877. (DOC) [file pone.0084078.s001.doc]

**Figure S1.** Extract of federal legislation in 1877

**Notes:** See BBl 1877 I 265, Botschaft des Bundesrathes an die hohe Bundesversammlung, betreffend die Frage, ob Vorlagen an das Schweizervolk mit einer erläuternden Botschaft zu begleiten seien [Message of the Federal Council addressed at the Federal Assembly regarding the question whether referenda to the Swiss sovereign need to be accompanied by an explanatory message] (February 14, 1877, Source: Schweizerisches Bundesarchiv). Constituents are generally well informed about parliamentary recommendations and the issues to be decided by referendum are explained in booklets/pamphlets. This practice was formally initiated in 1877 when the federal council argued for giving constituents an official publication detailing the legislative proposal submitted for the referendum. Parliament then entrusted the federal council with officially and objectively informing citizens on the reasons for the referendum and how parliament formed its opinion. The federal council’s address to parliament makes clear that, even in the decades before 1877, voters were informed by the media about parliament’s position, and as early as 1874, the federal council itself was disseminating such information (as stated in the Federal Gazette BBl 1874 I 497).
