# Supplementary material for: Bounded Rationality and Voting Decisions over 160 Years: Voter Behavior and Increasing Complexity in Decision-Making
Source: PLoS One. 2013 Dec 31;8(12):e84078. doi: 10.1371/journal.pone.0084078 (PMC3877213; doi:10.1371/journal.pone.0084078)
Supplement: Figure S2 — Total number of referenda from 1848 to 2009. (DOC) [file pone.0084078.s002.doc]

**Figure S2.** Total number of referenda from 1848 to 2009


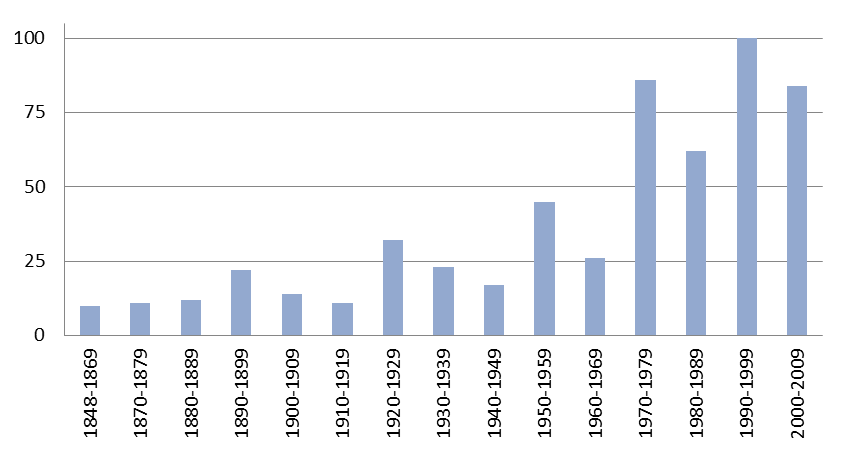


**Notes:** The figure summarizes the number of federal referenda per decade in our dataset from 1848 until the beginning of 2009. Note that voters may decide on cantonal and communal issues too, but they are clearly distinguishable from federal referenda and do not systematically take place in all cantons/communities together with federal referenda.
