# Supplementary material for: Bounded Rationality and Voting Decisions over 160 Years: Voter Behavior and Increasing Complexity in Decision-Making
Source: PLoS One. 2013 Dec 31;8(12):e84078. doi: 10.1371/journal.pone.0084078 (PMC3877213; doi:10.1371/journal.pone.0084078)
Supplement: Table S1 — Data description and sources. (DOC) [file pone.0084078.s003.doc]

**Table S1.** Data description and sources

| *Variable* | *Description & Source* |  | *1848-2009* | | *1848-1945* | | *1946-2009* | |
| --- | --- | --- | --- | --- | --- | --- | --- | --- |
|  |  | *Available since* | *Mean* | *SD* | *Mean* | *SD* | *Mean* | *SD* |
| Constituency accepts referendum | Indicator variable: Constituency (canton) accepts the referendum. Federal Statistical Office and Swissvotes Database. | 1848 | 0.4907 | 0.4999 | 0.4856 | 0.4999 | 0.4924 | 0.5000 |
| Parliament suggests YES | Indicator variable: Parliamentary majority suggests to vote YES. Federal Statistical Office and Swissvotes Database. | 1848 | 0.7031 | 0.4569 | 0.7887 | 0.4083 | 0.6743 | 0.4687 |
| More than one referendum | Indicator variable: More than one referendum on the same day. Author construction based on Swissvotes Database. | 1848 | 0.7334 | 0.4422 | 0.4718 | 0.4993 | 0.8212 | 0.3832 |
| More than two referenda | Indicator variable: More than two referenda on the same day. Author construction based on Swissvotes Database. | 1848 | 0.4692 | 0.4991 | 0.2042 | 0.4032 | 0.5582 | 0.4966 |
| More than three referenda | Indicator variable: More than three referenda on the same day. Author construction based on Swissvotes Database. | 1848 | 0.3001 | 0.4583 | 0.0916 | 0.2884 | 0.3701 | 0.4829 |
| Low turnout referendum | Indicator variable: Turnout in constituency is not highest when there is more than one referendum on the same day (takes a value of 0 by default when there is only one referendum on the same day). Author construction based on Swissvotes Database. | 1884 | 0.4822 | 0.4997 | 0.2492 | 0.4326 | 0.5466 | 0.4979 |
| Number of referenda on the same day | Number of referenda on the same day. Federal Statistical Office and Swissvotes Database. | 1848 | 2.8810 | 1.8827 | 2.0850 | 1.9629 | 3.1480 | 1.7768 |
| Counterproposal | Indicator variable: Referendum is a parliamentary counterproposal to an initiative. Federal Statistical Office and Swissvotes Database. | 1848 | 0.0631 | 0.2432 | 0.0493 | 0.2165 | 0.0678 | 0.2514 |
| Turnout | Number of valid votes in the constituency divided by number of eligible voters. Federal Statistical Office. | 1884 | 0.4615 | 0.1506 | 0.5526 | 0.1970 | 0.4363 | 0.1236 |
| Government neutral | Indicator variable: Government does not give a voting recommendation. Federal Statistical Office and Swissvotes Database. | 1848 | 0.2531 | 0.4348 | 0.4577 | 0.4983 | 0.1845 | 0.3879 |
| Interest groups divergent | Indicator variable: One or more major interest groups offer divergent voting recommendations. Swissvotes Database. | 1945 | 0.4816 | 0.4997 |  |  | 0.4828 | 0.4997 |
| All interest groups suggest YES | Indicator variable: All major interest groups suggest a YES vote. Swissvotes Database. | 1945 | 0.3679 | 0.4823 |  |  | 0.3664 | 0.4818 |

**Notes:** Unweighted descriptive statistics. Data sources indicated next to variable descriptions.
