# Supplementary material for: Bounded Rationality and Voting Decisions over 160 Years: Voter Behavior and Increasing Complexity in Decision-Making
Source: PLoS One. 2013 Dec 31;8(12):e84078. doi: 10.1371/journal.pone.0084078 (PMC3877213; doi:10.1371/journal.pone.0084078)
Supplement: Table S2 — Parliamentary influence on constituent referenda when government is neutral and members of parliament offer divergent suggestions. (DOC) [file pone.0084078.s004.doc]

**Table S2. Parliamentary influence on constituent referenda when government is neutral and members of parliament offer divergent suggestions**

|  | *Government neutral position* | | | *25% or more of members of parliament have diverging suggestion* | | |
| --- | --- | --- | --- | --- | --- | --- |
|  | *(1)* | *(2)* | *(3)* | *(4)* | *(5)* | *(6)* |
| Years covered | 1848-2009 | 1884-2009 | 1884-2009 | 1848-2009 | 1884-2009 | 1884-2009 |
| Parliament suggests YES | 1.3472*** (0.2986) | 1.3527*** (0.2999) | 2.4782*** (0.1274) | 0.7608 (0.5919) | 0.7511 (0.6034) | 1.9922*** (0.1685) |
| Parliament suggests YES * Number of referenda on the same day | **0.3940*** (0.0998)** | **0.3837*** (0.1003)** |  | **0.4946*** (0.1582)** | **0.4930*** (0.1640)** |  |
| Number of referenda on the same day | -0.2164** (0.0845) | -0.2189** (0.0855) | 0.0911*** (0.0145) | -0.2374** (0.1026) | -0.2351** (0.1023) | -0.0083 (0.0187) |
| Parliament suggests YES * Low turnout referendum |  |  | **0.2859** (0.1215)** |  |  | **0.9889*** (0.1576)** |
| Low turnout referendum |  |  | -0.0939 (0.0996) |  |  | -0.5107*** (0.1249) |
| Government neutral * Number of referenda on the same day | **-0.0472 (0.1372)** | **-0.0464 (0.1368)** |  |  |  |  |
| Government neutral * Low turnout referendum |  |  | **0.0067 (0.0805)** |  |  |  |
| Government neutral | 1.0361*** (0.3086) | 1.0119*** (0.3203) | 0.8751*** (0.0624) |  |  |  |
| Counterproposal | -0.4368 (0.3049) | -0.5058 (0.3080) | -0.5541*** (0.0540) | -0.4525 (0.6045) | -0.4753 (0.6057) | -0.4557*** (0.0682) |
| Turnout |  | -1.6868*** (0.4796) | -1.1955*** (0.2356) |  | -1.1502 (0.8319) | 0.0283 (0.4550) |
| Constituency Fixed Effects | YES | YES | YES | YES | YES | YES |
| Decade Fixed Effects | YES | YES | YES | YES | YES | YES |
| DE Parliament suggests YES | 40.76 | 40.80 | 52.59 | 29.72 | 29.50 | 43.04 |
| DE Parliament suggests YES * More than one referendum | 14.83 | 14.52 |  | 20.35 | 20.36 |  |
| DE Parliament suggests YES * Low turnout referendum |  |  | 5.22 |  |  | 16.80 |
| Clustering | Referendum | Referendum | Constituency | Referendum | Referendum | Constituency |
| Sample restriction |  |  | > 1 referendum |  |  | > 1 referendum |
| Pseudo R2 | 0.3512 | 0.3684 | 0.3792 | 0.3411 | 0.3541 | 0.3657 |
| Brier | 0.1813 | 0.1772 | 0.1741 | 0.1745 | 0.1719 | 0.1709 |
| No. Obs. | 14127 | 13502 | 9961 | 4379 | 4229 | 3698 |

**Notes:** The dependent variable for all logit estimations is *Constituency accepts referendum*. Robust standard error estimates are reported throughout the table and clusters are indicated. DE = discrete effect in the predicted probability (see Table 1 and text for details). ***, **, and * indicate a mean significance level of below 1 %, between 1 and 5 %, and between 5 and 10 %, respectively. In three cases during our sample period, there was disagreement between the government and parliament. Even if we exclude these three cases, however, our results do not change (not reported here). More important, in many cases, the government remains neutral and expresses no preference (no position), so we examine voter responses to such a situation to gauge whether constituents react to politicians’ suggestions in general or only to the government or parliament. Specification (1) uses a sample that begins in 1848 and includes an interaction term between government neutrality and the *Number of referenda on the same day*. The results entirely confirm our expectation that constituents will not react more to a neutral position (no voting recommendation) of the government if there is more than one referendum but will still react more to parliament’s position if the number of referenda increases: The interaction term between “Parliament suggests YES” and “Number of referenda on the same day” remains highly statistically significant and positive, with a large discrete effect (14.83 percentage points) while the interaction term for “Government neutral” and “Number of referenda on the same day” is both small and insignificant. Specification (2) employs a sample that starts in 1884 and includes turnout. The quantitative effects barely change for parliament’s recommendation and the interaction term for “government neutral” and “Number of referenda on the same day” remains insignificant. Finally, specification (3) analyzes those referenda that do not have the highest turnout, restricting the sample to days with more than one referendum. Again, the interaction term for the government is not statistically significant, but the interaction term for the parliamentary influence is highly significant and positive. It may also be that minorities in parliament or specific parties might try to set the referendum day in their own favor; that is, in such a way that their position has more power than the parliamentary majority. To take these potential strategic behaviors into account, we look at the majority by which parliament passed legislative proposals. The primary methodological problem is that systematic recording of the number of parliamentary members voting yes/no only began with the introduction of an electronic voting system in 1996, so for data before this, we had to refer to national council’s collected stenographic protocols and records of which politicians voted for and against a proposal. Stenographic protocols, however, particularly up until 1870 and between 1930 and 1945, do not always mention the exact majorities, only the final parliamentary decision. Hence, we have no information on the exact majorities in a total of 113 cases. To determine whether minority positions led the Federal Chancellery to strategically bundle referenda on the same day, specification (4) restricts the analysis only to recommendations on which 25% or more of members of parliament disagreed. Not only is the interaction term significant and positive but the discrete effect is higher than before. Thus, it is not the minorities who bundle/unbundle referenda on a single day; rather, they may only prevent the majority from strategically scheduling more referenda on the same day. This interpretation would explain why we now also find a higher effect of the influence of the parliamentary majority (i.e., parliamentary recommendation). Both specification (5), which uses data from 1884 onward, and specification (6), which analyzes referenda with low turnout (in line with specification (3)), also return a higher discrete effect.
