# Supplementary material for: Bounded Rationality and Voting Decisions over 160 Years: Voter Behavior and Increasing Complexity in Decision-Making
Source: PLoS One. 2013 Dec 31;8(12):e84078. doi: 10.1371/journal.pone.0084078 (PMC3877213; doi:10.1371/journal.pone.0084078)
Supplement: Table S3 — Robustness tests with different periods – Influence of Parliament on constituents’ choices in referenda. (DOC) [file pone.0084078.s005.doc]

**Table S3.** Robustness tests with different periods – Influence of Parliament on constituents’ choices in referenda

|  | *Without World War I and World War II* | | | *From 1946 to 2009* | | *From 1884 to 1945* | | |
| --- | --- | --- | --- | --- | --- | --- | --- | --- |
|  | *(1)* | *(2)* | *(3)* | *(4)* | *(5)* | *(6)* | *(7)* | *(8)* |
| Years covered | 1848-2009 | 1884-2009 | 1884-2009 | 1945-2009 | 1945-2009 | 1884-1945 | 1884-1945 | 1884-1945 |
| Parliament suggests YES | 1.7673*** (0.3054) | 1.7445*** (0.3070) | 2.5415*** (0.1230) | 1.9014*** (0.3645) | 2.5790*** (0.1287) | 1.0039*** (0.2438) | 2.0152*** (0.2363) | 1.6656*** (0.1702) |
| Parliament suggests YES * Number of referenda on the same day | **0.3061*** (0.0980)** | **0.3028*** (0.0989)** |  | **0.2781*** (0.1038)** |  | **0.5154*** (0.1130)** |  |  |
| Number of referenda on the same day | -0.1760** (0.0836) | -0.1802** (0.0850) | 0.0734*** (0.0143) | -0.1296 (0.0845) | 0.0853*** (0.0150) | -0.7457*** (0.0989) | -1.1675*** (0.1133) | -0.9466*** (0.1283) |
| Parliament suggests YES * Low turnout referendum |  |  | **0.2889** (0.1137)** |  | **0.3156*** (0.1162)** |  | **0.1186 (0.2419)** | **0.6062*** (0.1695)** |
| Low turnout referendum |  |  | -0.0468 (0.1011) |  | -0.0129 (0.1019) |  | -0.3216 (0.2323) | -0.7438*** (0.1652) |
| Exactly one referendum per day |  |  |  |  |  |  |  | -1.3179*** (0.1752) |
| Counterproposal | -0.3357 (0.3065) | -0.4189 (0.3117) | -0.4604*** (0.0535) | -0.5553* (0.3258) | -0.5050*** (0.0589) | 1.0161*** (0.1497) | 0.7162*** (0.1896) | 1.0211*** (0.1519) |
| Turnout |  | -1.9534*** (0.4719) | -1.4116*** (0.2448) | -1.9834*** (0.5843) | -1.7267*** (0.3806) | -2.4131*** (0.3073) | -1.0739** (0.5206) | -2.0597*** (0.3105) |
| Constituency Fixed Effects | YES | YES | YES | YES | YES | YES | YES | YES |
| Decade Fixed Effects | YES | YES | YES | YES | YES | YES | YES | YES |
| DE Parliament suggests YES | 47.18 | 46.97 | 53.40 | 48.89 | 53.48 | 36.22 | 44.48 | 39.37 |
| DE Parliament suggests YES * More than one referendum | 10.71 | 10.72 |  | 9.82 |  | 10.58 |  |  |
| DE Parliament suggests YES * Low turnout referendum |  |  | 5.63 |  | 6.51 |  | 3.89 | 9.80 |
| Clustering | referendum | referendum | constituency | referendum | constituency | constituency | constituency | constituency |
| Sample restriction |  |  | > 1 referendum |  | > 1 referendum |  | > 1 referendum |  |
| Pseudo R2 | 0.3294 | 0.3487 | 0.362 | 0.3815 | 0.3801 | 0.2947 | 0.3653 | 0.3079 |
| Brier | 0.1856 | 0.1813 | 0.1776 | 0.1741 | 0.1734 | 0.1904 | 0.1781 | 0.1882 |
| No. Obs. | 13652 | 13027 | 9861 | 10577 | 8686 | 2925 | 1275 | 2925 |

**Notes:** The dependent variable for all logit estimations is *Constituency accepts referendum*. Robust standard error estimates are reported throughout the table and clusters are indicated. DE = discrete effect in the predicted probability (see Table 1 and text for details). ***, **, and * indicate a mean significance level of below 1 %, between 1 and 5 %, and between 5 and 10 %, respectively. In specifications (1) to (3), we exclude World War I and II, as those years represent exceptional circumstances. Such exclusion, however, has no effect on our key results. In specifications (4) to (5), we look at a more recent time period (1946 to 2009), a very stable era in Switzerland with good economic growth and increased wealth. Although all interaction terms are significant, the discrete effect in specification (4) is relatively smaller, most likely due to the post-1945 tendency to hold more referenda on one day. Yet this finding also implies that the effect of the interaction with “low turnout referendum” should become more important, which is precisely the case—the discrete effect is 6.51 percentage points compared to around 5 percentage points in earlier estimates. Specifications (6) to (8) focus on the period up until 1945. Specification (6) produces the now typical result: the discrete effect is again higher because of a reference group with a sufficient number of observations. In specification (7), however, for the first time, the interaction effect between “Parliament suggests YES” and “Low turnout referendum” is not statistically significant. We assume two possible reasons for this result: (1) the sample is comparatively small or (2) the instances of two or more referenda before 1945 are few, so low turnout referenda are identified mostly on days with two referenda. We solve this bias in specification (8) by controlling for days with only one referendum.
