# Supplementary material for: Bounded Rationality and Voting Decisions over 160 Years: Voter Behavior and Increasing Complexity in Decision-Making
Source: PLoS One. 2013 Dec 31;8(12):e84078. doi: 10.1371/journal.pone.0084078 (PMC3877213; doi:10.1371/journal.pone.0084078)
Supplement: Table S4 — Rolling regression – Parliamentary influence on constituent referenda choices. (DOC) [file pone.0084078.s006.doc]

**Table S4.** Rolling regression – Parliamentary influence on constituent referenda choices

| *Excluded constituency* | *Reported variable: (Parliament suggests YES) * (Number of referenda on the same day)* | |
| --- | --- | --- |
|  | *Coefficient* | *Standard error* |
| AG | 0.3019*** | 0.096 |
| AI | 0.3039*** | 0.096 |
| AR | 0.3063*** | 0.096 |
| BE | 0.3099*** | 0.096 |
| BL | 0.3069*** | 0.096 |
| BS | 0.3138*** | 0.099 |
| FR | 0.3041*** | 0.097 |
| GE | 0.3225*** | 0.101 |
| GL | 0.3069*** | 0.096 |
| GR | 0.3220*** | 0.096 |
| JU | 0.3205*** | 0.099 |
| LU | 0.3070*** | 0.095 |
| NE | 0.3125*** | 0.099 |
| NW | 0.3039*** | 0.096 |
| OW | 0.3099*** | 0.096 |
| SG | 0.3087*** | 0.096 |
| SH | 0.3109*** | 0.096 |
| SO | 0.3028*** | 0.095 |
| SZ | 0.3111*** | 0.096 |
| TG | 0.3102*** | 0.096 |
| TI | 0.3143*** | 0.097 |
| UR | 0.3153*** | 0.096 |
| VD | 0.3168*** | 0.098 |
| VS | 0.3162*** | 0.097 |
| ZG | 0.3072*** | 0.095 |
| ZH | 0.3037*** | 0.095 |

**Notes:** The dependent variable for all logit estimations is *Constituency accepts referendum*. The first column indicates the constituency which was excluded when estimating specification (2) of Table 1. ***, **, and * indicate a mean significance level of below 1 %, between 1 and 5 %, and between 5 and 10 %, respectively.
